# THINK Back: KNowledge-based Interpretation of High Throughput data

**DOI:** 10.1186/1471-2105-13-S2-S4

**Published:** 2012-03-13

**Authors:** Fernando Farfán, Jun Ma, Maureen A Sartor, George Michailidis, Hosagrahar V Jagadish

**Affiliations:** 1 Computer Science and Engineering Department, University of Michigan, Ann Arbor, MI, USA; 2 Department of Molecular and Human Genetics, Baylor College of Medicine, Houston, TX, USA; 3 Center for Computational Medicine and Bioinformatics, University of Michigan, Ann Arbor, MI, USA; 4 Department of Statistics, University of Michigan, Ann Arbor, MI, USA

## Abstract

Results of high throughput experiments can be challenging to interpret. Current approaches have relied on bulk processing the set of expression levels, in conjunction with easily obtained external evidence, such as co-occurrence. While such techniques can be used to reason probabilistically, they are not designed to shed light on what any individual gene, or a network of genes acting together, may be doing. Our belief is that today we have the information extraction ability and the computational power to perform more sophisticated analyses that consider the individual situation of each gene. The use of such techniques should lead to qualitatively superior results.

The specific aim of this project is to develop computational techniques to generate a small number of biologically meaningful hypotheses based on observed results from high throughput microarray experiments, gene sequences, and next-generation sequences. Through the use of relevant known biomedical knowledge, as represented in published literature and public databases, we can generate meaningful hypotheses that will aide biologists to interpret their experimental data.

We are currently developing novel approaches that exploit the rich information encapsulated in biological pathway graphs. Our methods perform a thorough and rigorous analysis of biological pathways, using complex factors such as the topology of the pathway graph and the frequency in which genes appear on different pathways, to provide more meaningful hypotheses to describe the biological phenomena captured by high throughput experiments, when compared to other existing methods that only consider partial information captured by biological pathways.

## Background

Microarray experimental data are used extensively to profile not only the expression levels of thousands of genes simultaneously [1], but also DNA methylation levels and transcription factor binding across the promoters of thousands of genes. The data obtained from these experiments are often used to study gene functions and interactions within biological pathways. These experiments produce a myriad of data and the results for individual genes are often not reproducible [2,3]. As such, the process of generating biological hypotheses from such experiments is often very complex.

The invention of new computational methods has allowed the analysis of experimental microarray data to evolve from single-gene analysis techniques [4-6], to group testing procedures [7-9]. These methods compare either the set of significantly-changed genes within a microarray experiment or some measure of significance for all genes in a microarray experiment against previously defined lists of genes that represent a biological phenomenon or concept (e.g. biological pathways, Gene Ontology [GO] categories [10]). [9,11] survey this topic in detail. In our previous work [12,13], we proposed a model-based approach for testing the significance of biological pathways using the underlying gene network and studied graph theoretic properties of the model. Also, our GPCR [14] method performs a dimension reduction over the pathway graph, with the sub-networks of interest defined *a priori*.

Even though the development of these computational methods represents a leap forward towards achieving a more robust analysis of high-throughput data, we observe that many of these methods apply only limited biological knowledge to the analytical process. The goal of this project is to emulate computationally, for thousands of candidate genes, what a biomedical scientist would want to do for one gene. This means bringing to bear as much biological knowledge as possible, as found in the literature and in public databases, to develop biologically sound hypotheses that could explain the observed differential expression.

With this in mind, we have devised THINK-Back: KNowledge-based Interpretation of High Throughput data. Our objective is to develop a suite of computational tools and methods that generate a small number of biologically meaningful hypotheses based on observed results from high throughput experiments, through the use of relevant known biomedical knowledge, as presented in pathway databases, gene interaction networks and other sources of knowledge. The THINK-Back suite provides a set of tools for the analysis of microarray data that are both robust yet easy to use.

In this paper we describe two methods to perform robust analysis of microarray data by exploiting the knowledge captured in biological pathway databases, such as the Kyoto Encyclopedia of Genes and Genomes (KEGG) [15], Protein ANalysis THrough Evolutionary Relationships (PANTHER) [16], Reactome [17], GenMapp [18], and Biocarta http://www.biocarta.com. These enrichment testing methods have been published as a suite of Web services for public use. We briefly describe these web services in the following sections.

## Methods

The THINK-Back suite is a set of tools that provide a robust gene set enrichment testing analysis of microarray data, using pathways as a source of biological knowledge. The goal of these tools is to derive high-quality hypotheses regarding microarray data. To do so, each of these tools performs a complex and specific analysis over the biological pathway database.

Our analysis methods are further used to adjust the scores of previously developed methods (e.g. GSA, GSEA and LRpath). Our methods compute a score for each studied pathway and that score is used to adjust the score produced by the underlying group testing technique for the same pathway. A weight for each pathway is computed based on our score, which serve as *p*-value weights and hence need to be transformed to ensure that they are positive and that they increase with increasing levels of differential expression, that is, they must be positively correlated with increasing importance. Table 1 summarizes the methods we have developed as well as how we have implemented them as adjustment factors to previously-developed methods.

**Table 1:** Summary of THINK-Back tools and adjustment methods

THINK-Back Method	**Abbr**.	Adjustment to prior methods			
		**GSEA **[19]	**GSA **[24]	**LRpath **[23]	

Gene Appearance Frequency	AF	GSEA-AF	-	LRpath-AF	
Density Analysis	DS	GSEA-DS	GSA-DS	LRpath-DS	

Our two THINK-Back adjustment methods can be used in combination with previously-developed gene set enrichment tools.

### Gene Appearance Frequency Analysis

Gene set enrichment testing has helped to close the gap from an individual gene to a systems biology interpretation of microarray data. Unfortunately, although gene sets are defined *a priori *based on biological knowledge, current methods for gene set enrichment testing treat all genes equal. It is well known that some genes, such as those responsible for housekeeping functions, appear in many pathways, whereas other genes are more specialized and play a unique role in a single pathway.

Drawing inspiration from the field of Information Retrieval (IR), we have developed an approach to incorporate the frequency in which a specific gene appears in a pathway. We then use the results of this analysis to adjust previously-developed group testing techniques, such as GSEA and LRpath, to generate more reproducible and biologically meaningful results.

For example, in GSEA [19], genes are first ranked by a signal to noise ratio. A "running sum" statistic is calculated for each gene set, based on the ranks of members in the set, relative to those of non-members. An enrichment score (ES) is defined to be the maximum of the running sum across all genes, which corresponds to a weighted Kolomogorov-Smirnov statistic. The weight places more importance on the top and bottom of the ranked list. When a gene set contains a large number of highly ranked genes, a high ES is achieved.

In the original implementation of GSEA, the running-sum statistics used equal weights at every step. Our Gene Frequency Appearance technique [20] adopts a classical Information Retrieval term weighting method [21]. The importance of a term in a given document can be estimated by multiplying the raw *term frequency *(TF) of the term in a document by the term's *inverse document frequency *(IDF) weight. The importance increases proportionally to the number of times a word appears in the document but is offset by the frequency of the word in a collection. This method was easily transformed for our purpose. Each pathway map is composed of a group of genes, which is the analog of a document and the words in it.

Since most genes only appear once in a given pathway map, the term frequency does not provide further useful information in our case. The inverse term frequency is a measure of general importance of the term. IDF is obtained by dividing the number of all documents by the number of documents containing the term, and then taking the logarithm. In our case, the number of documents containing the term is the appearance frequency of genes across all KEGG pathways.

### Density Analysis

As mentioned earlier, there are several methods that use biological pathways to interpret microarray experiments. Nevertheless, the biological knowledge captured by the pathway networks is typically introduced in a very simple way--for example all genes in the pathway are defined to be in a gene set and considered equivalent for statistical purposes. Our Density Analysis Score (DS) method takes into account the topology of the pathway graph by considering the relative positions of differentially expressed genes over the pathway network. This score is then used to adjust any prior gene set enrichment testing scores.

**Figure 1:**
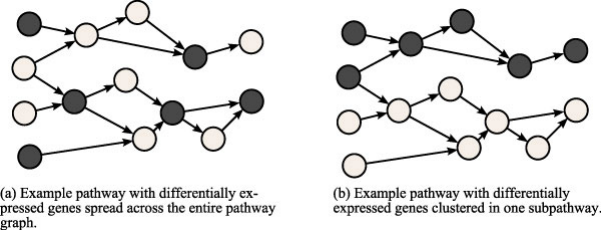
**Example of density analysis on biological pathways**. Two example pathways with differentially expressed genes appearing in different configurations. A pathway with differentially expressed genes appearing tightly-clustered in one portion of the graph is more significant than a pathway in which the differentially expressed genes appear spread out.

Our assumption for the method proposed here is that a pathway with a closely-connected cluster of differentially expressed genes is more likely informative and relevant than a pathway which has the same number of differentially expressed genes spread out uniformly or randomly across the pathway. Figure 1 illustrates this idea intuitively: it presents two different configurations for an example pathway. Figure 1(a) shows differentially expressed genes spread out uniformly across the pathway; in contrast, Figure 1(b) shows the same number of differentially expressed genes, but clustered in one portion of the pathway, creating a tight cluster of connected genes. We can observe how the pathway is more clearly activated in Figure 1(b) than in Figure 1(a). We justify this assumption by observing that since pathways are often activated via sub-paths, one does not expect the expression levels of all genes to change in an activated pathway. This is partially because the activity level of some genes may change through a different mechanism, but also because some canonical pathways are defined in ways that involve more than one function. For example, the KEGG pathway for "Apoptosis" involves a sub-path leading to apoptosis and a sub-path leading to cell survival.

To achieve our objective, we create a graph representation of each pathway. We let the nodes in the graph represent the genes in the pathway and the edges between nodes represent the interactions between genes. We then calculate the pair-wise shortest paths from each gene in the pathway graph to every other gene in the graph. The Floyd-Warshall algorithm can be used to compute this in Θ(*n*^3^) time complexity [22].

We then compute the density score *ds *for all the genes in the pathway to represent the effect of one gene over another in the sub-graph with a penalty of the distance between the genes. It signifies the effect of global differential expression values on a local site by giving higher significance to closely clustered differentially expressed genes. The final score for the pathway is calculated by computing the average of the density scores across all genes in the pathway. This final score favors both the ratio of differentially expressed genes within the pathway, as well as the distance between the differentially expressed genes and the relative position among them. Pathways are ranked in decreasing order of their density score values. The pathways that have higher density score are deemed more significant than the pathways that have lower density score.

## Results and discussion

We have developed and deployed a suite of gene set enrichment testing tools that provide a richer analysis than the state-of-the-art tools, by applying a complex analysis of KEGG pathways and exploiting some factors that can lead to a better application of the underlying biological knowledge.

### Gene Appearance Frequency Analysis

**Figure 2:**
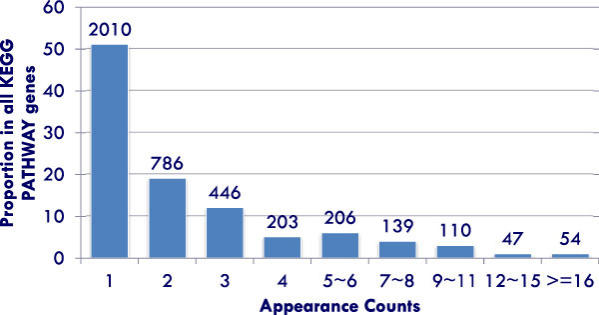
**Distribution of appearance frequency of genes in KEGG Pathway database**. The *x *axis indicates the appearance frequency of each gene in KEGG Pathways. The *y *axis shows the proportion of genes with indicated appearance frequency out of all KEGG Pathway genes. The total number of genes at each appearance count is indicated.

When applying the Gene Appearance Frequency (AF) method described in the previous section over KEGG pathways, we can confirm our assumptions regarding the varying appearance frequency of genes and to explore the biological basis for the observed variance. Figure 2 shows the distribution of appearance frequency of genes within KEGG pathways. Half of the genes appear only once in a specific pathway.

These genes are evenly distributed among all KEGG pathways, without significant enrichment in any particular gene set. A decreasing proportion of genes have an increasing frequency of appearance. Less than two percent of KEGG pathway genes appear more than sixteen times. This figure reflects the underlying biology of signaling pathways and the property of gene occurrence within them.

We applied the GSEA and GSEA-AF methods on two independent breast cancer datasets, which were originally analyzed and compared in [23]. GSEA-AF identified more overlapping KEGG pathways than GSEA. Examining the overlapping gene sets with False Discovery Rate *FDR <*= 0.05 in the ranked list generated by GSEA and GSEA-AF (Table 2), we see that there are more overlapping gene sets discovered by GSEA-AF. More specifically, one more breast cancer related gene set was identified by GSEA-AF. For a detailed experimental evaluation of this technique, please see [20].

**Table 2:** Comparison of overlapping gene sets generated by GSEA and GSEA-AF

Methods	Overlap Gene Sets (*FDR <*0.05)	Cancer Related Gene Sets	Name of Gene Sets
GSEA	7	3	Proteasome, Cell cycle, Biosynthesis of steroids
GSEA-AF	9	4	Proteasome, Cell cycle, Biosynthesis of steroids, Oxidative phosphorylation

The ranked list generated by GSEA was ranked by Normalized Enrichment Score (NES). Only gene sets with qvalue less than 0.05 were considered.

### Density Analysis

We also executed our Density Score analysis with the breast cancer datasets described earlier. We ran both the standard methods and the DS-adjusted methods with KEGG pathways on the paired data sets and ranked the pathways based on their descending order of significance. We then calculated the correlation coefficient of the enrichment scores between paired data sets, and the correlation coefficient of the ranks of the gene sets between paired data sets in order to compare the results. We focus our evaluation on a set of signaling pathways that have been identified as genetically altered in a majority of cancers.

Similar to our experiment with AF, we appear to find more biologically relevant results with the DS-adjusted methods. Table 3 presents the summary of improvement in rankings for 19 cancer-related KEGG signaling pathways, when using the DS-adjusted methods. In more detail, we observe that in GSEA-DS, "Toll-like receptor signaling pathway" and "Wnt signaling pathway" are ranked in the top pathways. The Wnt pathway is another conserved pathway critical for mammalian development and adult tissue maintenance and hyper-activated in most human cancers. "Apoptosis" and "p53 signaling pathway", two other cancer-related pathways, are also ranked higher in GSEA-DS.

**Table 3:** Ranking improvement for cancer-related KEGG signaling pathways with DS

Dataset	#Pways	GSA-DS			LRpath-DS			GSEA-DS		
		
		0 - 10	*>*10	TOTAL	0 - 10	*>*10	TOTAL	0 - 10	*>*10	TOTAL
Breast GSE-2990	18	8	5	13	7	8	15	6	7	13
Breast GSE-3494	18	9	5	14	8	5	13	10	5	15
Lung Boston	18	4	4	8	4	9	13	5	6	11
Lung Michigan	18	6	7	13	4	6	10	7	7	14

**AVERAGE**		**7**	**5**	**12**	**6**	**7**	**13**	**7**	**6**	**13**

This table presents the summary of improvement in rankings for 19 cancer-related KEGG signaling pathways, when using the DS-adjusted methods. We can observe that out of these 19 cancer-related pathways, DS-adjusted methods improve the rankings of more than half of the pathways, with LRpath-DS showing average improvements in the rankings of 78% of the mentioned signaling pathways. We also differentiate between large ranking improvements (greater than 10 ranks), and observe that GSEA-DS has an average rank improvement greater than ten ranks in 69% of the studied pathways.

### THINK-Back web services

The main objective of the THINK-Back suite is to develop and deploy a series of gene set enrichment testing methods that can be used by scientists worldwide. To achieve this goal, we have implemented the computational tools described in the previous section and have made them available as web services. Extensive documentation and examples for the THINK-Back web services can be accessed at http://www.eecs.umich.edu/db/think/software.html.

### THINK-Back software architecture

**Figure 3:**
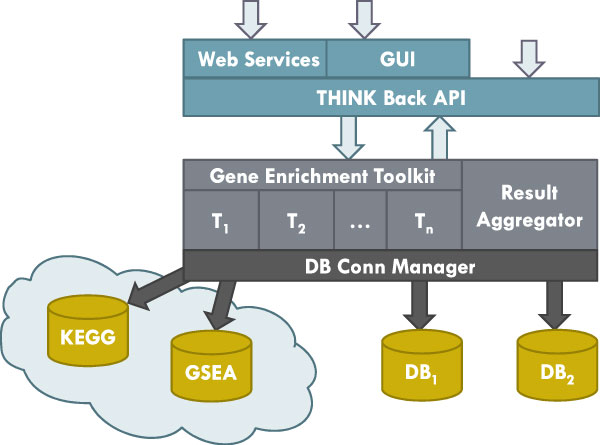
**THINK-Back Software Architecture**. The THINK-Back suite of tools has been developed to allow scientists worldwide to use our gene set enrichment testing methods and to combine them with previously-developed methods as adjustment tools. These tools can be accessed via web services, an application programmer's interface (API), and soon we will be providing a web-based user interface as well.

We have implemented our THINK-Back suite of tools under the software architecture depicted in Figure 3. This architecture allows us to easily connect to several pathway databases (e.g. KEGG, PANTHER) to utilize that data for our analysis. In addition, it provides a transparent mechanism to publish interfaces to our methods, via web services, an application programmer's interface (API), and soon we will be providing a web-based user interface as well.

### Web service usage

**Figure 4:**
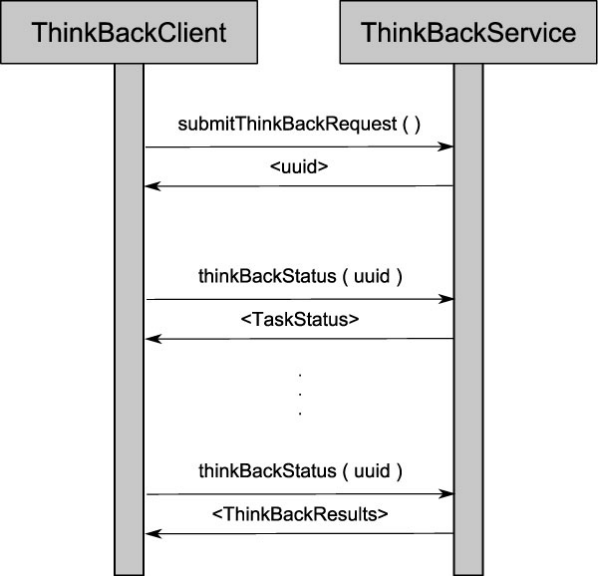
**THINK-Back Web Service Call Diagram**. The sequence diagram shows the steps to call the THINK-Back Web service, request the job status, and finally obtain the results. After submitting the job for execution, the web service returns a unique identifier that should be used to check the job status. Once the status returned is DONE, the user can check for the results of the execution.

The THINK-Back web services have been implemented with the purpose of enabling scientists to access our tools from anywhere in the world, and have been designed to be executed as long-running tasks. This means that the service can be invoked and will return the results asynchronously. These web services have been included into the suite of web Long Running Web Services of the National Center for Integrative Biomedical Informatics (NCIBI), available at http://ws.ncibi.org/longrunning.html. When the web service is initially invoked, the request is sent to an execution queue and a unique identifier for the job is returned. The user can then check the status of the desired job until it is completed. Once completed, the web service returns the list of results, including the pathway identifier and its p-value. Figure 4 describes this process. We have provided a sample Java implementation of the THINK-Back client in Additional File 1 . The example shows the client class submitting a request to the web service to perform the GSEA-DS enrichment method over the breast cancer sample described in the Results Section. After the web service is invoked and the unique identifier is obtained, the client proceeds to poll for the job to be done, checking the status every ten minutes. Once the DONE status is received, the user can check the results of the enrichment method, having the adjusted p-value for each analyzed pathway.

We have deployed all necessary Java classes in a Maven http://maven.apache.org/ repository. A project object model (POM) file containing all the required references to build the Java project is also included in Additional File 2.

## Competing interests

None declared.

## Authors' contributions

The initial idea was conceived in discussions between all five authors. The code implementation was conducted by FF and JM, with the advice of the other authors. The initial manuscript draft was prepared by FF and JM, and then substantially edited by the other authors. All authors have read and approved the final manuscript.

## Supplementary Material

Additional file 1 **Java implementation of the THINK-Back client (NcibiLongRunningSample3.java)**. This Java class shows the execution of a request to the web service to perform the GSEA-DS enrichment method over the breast cancer sample (GEO Accession number GSE3494) described in the Results Section of the paper. Click here for file

Additional file 2 **THINK-Back Project Object Model (POM) file**. We have deployed all necessary Java classes in a Maven repository. This project object model (POM) file contains all the required references to build the Java project to invoke the THINK-Back web services. Click here for file

## References

[B1] Brown P Botstein D Exploring the new world of the genome with DNA microarrays Nature Genetics 1999 21 1 Suppl 33 37 9915498 10.1038/4462

[B2] Larkin J Frank B Gavras H Sultana R Quackenbush J Independence and reproducibility across microarray platforms Nature Methods 2005 2 5 337 344 10.1038/nmeth757 15846360

[B3] Draghici S Khatri P Eklund A Szallasi Z Reliability and reproducibility issues in DNA microarray measurements TRENDS in Genetics 2006 22 2 101 109 10.1016/j.tig.2005.12.005 16380191 PMC2386979

[B4] Tusher V Tibshirani R Chu G Significance analysis of microarrays applied to the ionizing radiation response Proc Natl Acad Sci U S A 2001 98 9 5116 5121 10.1073/pnas.091062498 11309499 PMC33173

[B5] Smyth GK Linear models and empirical Bayes methods for assessing differential expression in microarray experiments Stat Appl Genet Mol Biol 2004 3 Article 3 10.2202/1544-6115.1027 16646809

[B6] Sartor M Tomlinson C Wesselkamper S Sivaganesan S Leikauf G Medvedovic M Intensity-based hierarchical Bayes method improves testing for differentially expressed genes in microarray experiments BMC Bioinformatics 2006 7 538 10.1186/1471-2105-7-538 17177995 PMC1781470

[B7] Khatri P Draghici S Ostermeier G Krawetz S Profiling gene expression using onto-express Genomics 2002 79 2 266 270 10.1006/geno.2002.6698 11829497

[B8] Curtis R Oresic M Vidal-Puig A Pathways to the analysis of microarray data TRENDS in Biotechnology 2005 23 8 429 435 10.1016/j.tibtech.2005.05.011 15950303

[B9] Manoli T Gretz N Gröne H Kenzelmann M Eils R Brors B Group testing for pathway analysis improves comparability of different microarray datasets Bioinformatics 2006 22 20 2500 10.1093/bioinformatics/btl424 16895928

[B10] Ashburner M Ball C Blake J Botstein D Butler H Cherry J Davis A Dolinski K Dwight S Eppig J Gene Ontology: tool for the unification of biology Nature Genetics 2000 25 25 29 10.1038/75556 10802651 PMC3037419

[B11] Goeman J Bühlmann P Analyzing gene expression data in terms of gene sets: methodological issues Bioinformatics 2007 23 8 980 10.1093/bioinformatics/btm051 17303618

[B12] Shojaie A Michailidis G Analysis of gene sets based on the underlying regulatory network J Comput Biol 2009 16 3 407 426 10.1089/cmb.2008.0081 19254181 PMC3131840

[B13] Shojaie A Michailidis G Network enrichment analysis in complex experiments Stat Appl Genet Mol Biol 2010 9 Article 22 10.2202/1544-6115.1483 PMC2898649 20597848

[B14] Shojaie A Michailidis G Lafferty J, Williams C, Shawe-Taylor J, Zemel R, Culotta A Penalized principal component regression on graphs for analysis of subnetworks Advances in Neural Information Processing Systems 2010 23 2155 2163

[B15] Kanehisa M Goto S KEGG: Kyoto encyclopedia of genes and genomes Nucleic Acids Research 2000 28 27 10.1093/nar/28.1.27 10592173 PMC102409

[B16] Thomas P Campbell M Kejariwal A Mi H Karlak B Daverman R Diemer K Muruganujan A Narechania A PANTHER: a library of protein families and subfamilies indexed by function Genome Research 2003 13 9 2129 10.1101/gr.772403 12952881 PMC403709

[B17] Joshi-Tope G Gillespie M Vastrik I D'Eustachio P Schmidt E de Bono B Jassal B Gopinath GR Wu GR Matthews L Reactome: a knowledgebase of biological pathways Nucleic Acids Res 2005 33 Database issue D428 D432 15608231 10.1093/nar/gki072 PMC540026

[B18] Dahlquist K Salomonis N Vranizan K Lawlor S Conklin B GenMAPP, a new tool for viewing and analyzing microarray data on biological pathways Nature Genetics 2002 31 19 20 10.1038/ng0502-19 11984561

[B19] Subramanian A Tamayo P Mootha VK Mukherjee S Ebert BL Gillette MA Paulovich A Pomeroy SL Golub TR Lander ES Gene set enrichment analysis: a knowledge-based approach for interpreting genome-wide expression profiles Proc Natl Acad Sci U S A 2005 102 43 15545 15550 10.1073/pnas.0506580102 16199517 PMC1239896

[B20] Ma J Sartor M Jagadish H Appearance frequency modulated gene set enrichment testing BMC Bioinformatics 2011 12 81 10.1186/1471-2105-12-81 21418606 PMC3213687

[B21] Salton G Buckley C Term-weighting approaches in automatic text retrieval Information Processing and Management: an International Journal 1988 24 5 513 523 10.1016/0306-4573(88)90021-0

[B22] Floyd R Algorithm 97: shortest path Communications of the ACM 1962 5 6 345

[B23] Sartor M Leikauf G Medvedovic M LRpath: a logistic regression approach for identifying enriched biological groups in gene expression data Bioinformatics 2009 25 2 211 10.1093/bioinformatics/btn592 19038984 PMC2639007

[B24] Efron B Tibshirani R On testing the significance of sets of genes The Annals of Applied Statistics 2007 1 107 129 10.1214/07-AOAS101

